# Change in Patient Perceptions of Electronic Communication Methods for an Orthopaedic Fracture Clinic Between 2019 and 2024

**DOI:** 10.7759/cureus.74722

**Published:** 2024-11-29

**Authors:** Siddhartha Murhekar, Ali Soffar, Abdullah Khawaja, Aman Patel, Haritha Haridas Mandoth Veetil, Keval Patel, Thomas L Lewis

**Affiliations:** 1 Trauma and Orthopaedics, Princess Royal University Hospital, King's College National Health Service (NHS) Foundation Trust, London, GBR; 2 Trauma and Orthopaedics, King’s College London (Guy's, King's College and St Thomas' Hospitals) School of Medical Education, London, GBR

**Keywords:** communication preferences, digital communication, healthcare technology, healthcare technology adoption, missed appointments, missed appointments reduction, orthopaedic outpatient communication, outpatient attendance, patient satisfaction with communication methods, privacy concerns in medical communication

## Abstract

Introduction

Outpatient appointments are essential to patient care, yet rising non-attendance rates (‘did not attend’ DNAs) pose significant challenges, costing the National Health Service (NHS) over £1 billion annually. Traditional postal communication is inefficient and costly, while digital methods like short messaging service (SMS) and electronic mail (email) show the potential to improve attendance and reduce costs. This study investigates changes in patient preferences for orthopaedic outpatient communication methods between 2019 and 2024 at a busy district general hospital in London, UK.

Methods

A comparative cross-sectional study was conducted in 2019 and repeated in 2024. A total of 202 participants were involved in this study. A paper questionnaire was administered during fracture clinic visits, collecting data on electronic communication preferences, technology access, and missed appointments.

Results

The proportion of participants preferring email communication increased significantly from 68.89% in 2019 to 80.61% in 2024 (p < 0.05). Smartphone ownership also significantly rose from 66.32% to 84.46% (p < 0.05). There were no significant differences in satisfaction with other-based communication across cohorts, but an increasing trend towards digital communication was noted (p > 0.05).

Conclusion

Patients show a preference for digital communication over traditional methods. Adopting email-based systems could potentially improve attendance and reduce missed appointments. Further research is needed to address privacy concerns and optimise communication based on patient preferences.

## Introduction

Outpatient appointments are an integral and ever-growing part of patient care. In the National Health Service (NHS), outpatient appointments increased from 63.2 million in 2006-2007 to 135.4 million in 2023-2024, with an increase of 8.8% from 2022-2023 [1]. However, the attendance rate for all outpatient appointments has dropped from 80.6% in 2013-2014 to 77.3% in 2023-2024 [1]. A significant concern is the rise in appointments where the patient failed to turn up without any warning, also known as ‘did not attend’ (DNA). DNAs have risen by 4.2% between 2019-2020 and 2023-2024 [1]. Trauma and Orthopaedics has one of the highest DNA rates of any speciality at 7.2% [1].

Failed attendance costs the NHS £153 per appointment, with an annual reported cost of >£1 billion nationally [2]. Furthermore, the opportunity cost of treating other patients to minimise delays to care and waiting lists for appointments must be considered. When considering strategies to minimise the detrimental effects of DNAs, it is crucial to consider the method of communication. Outpatient scheduling is traditionally communicated to patients through postal letters in the NHS. Reliance on paper messages is a slow and non-environmentally friendly method of communication in the digital era. Furthermore, letters themselves have a significant cost associated with them. In 2016/2017, Guy’s and St Thomas’ NHS Foundation Trust reported a financial benefit of 2.6 million pounds and a reduction in the DNA rate of 17% by using digital outpatient communication systems such as DrDoctor (DrDoctor, London, UK) [2].

The use of technology should be considered to increase outpatient attendance whilst reducing resource use and detrimental impact on the environment in the NHS. Literature suggests that using a low-cost short messaging service (SMS) in a healthcare setting could be an effective strategy to minimise the rate of DNAs [3,4]. Furthermore, multiple SMS notifications before an outpatient appointment have been shown to improve attendance further [5]. Alternative digital communication, such as electronic mail (email), has shown similar, positive potential in healthcare communications [6]. Plener et al. demonstrated favourable use of email with patients and clinicians alike in the primary care setting [7]. Harnessing technology must be integral in modern healthcare to reduce the financial burden and economic impact of postal communication whilst attempting to improve attendance.

The widespread use of the internet, email and smartphones is central to our daily communication methods. In 2020, 96% of all households in Great Britain had access to the Internet, up from 93% in 2019, whilst 80% of households with one adult aged 65 years and over had access to the Internet [8,9]. Email is the most common internet activity used by 85% of adults in Great Britain in 2020, suggesting the central role it could play in communication for the NHS [8]. This is pertinent to DNAs as an analysis of the NHS's outpatient activity shows that those aged 30-39 had the highest proportion of the total appointments that were not attended, at 19.09% [1]. Attendance rates may increase through repeated communication through a patient's preferred method of communication. Furthermore, communication with minimal latency, such as email, may connect clinicians to patients more readily, which is an important consideration in the COVID-19 era and beyond.

The rapid adoption of technology brings the opportunity to improve healthcare communication for the benefit of patients, clinicians and institutions. The objectives of the present study are to explore patient perspectives regarding preferences of healthcare communication to further inform hospital practice and how they changed from 2019 to 2024.

## Materials and methods

Study design

A cross-sectional comparative study of patients attending outpatient orthopaedic fracture clinic appointments in a single district general hospital in the southeast United Kingdom was undertaken. The inclusion criteria for this study were any patient aged 16 and over who had attended the fracture clinic for an appointment and voluntarily took part where offered. 

Setting and participants

Participants undertook a questionnaire (Appendix) between May and June of 2019 in the first cohort and July to August 2024 in the second cohort when attending outpatient fracture clinic appointments. The fracture clinic uses a combination of postal letters and SMS scheduling of appointments.

Patients voluntarily completed a questionnaire regarding their communication preferences. The completed questionnaire was anonymous and entered into a collection box before their clinical encounter for later processing. The study did not require ethical approval as it was classified as a service evaluation and registered accordingly. The data was divided into two cohorts: the ‘2019 cohort’ and the ‘2024 cohort’.

Bias 

In an attempt to minimise bias, all participants were anonymised, and data analysts were blinded to responses. 

Statistical analysis

Continuous data were analysed with independent t-tests. Comparative analysis was performed using descriptive statistics and chi-squared testing. All statistical analyses were performed using the Python SciPy package (Travis Oliphant, Pearu Peterson and Eric Jones, Austin, TX) [10]. Statistical significance was defined as a p-value of less than 0.05.

## Results

A total of 202 participants were included during the study period. In the first cohort of 2019, there were 99 patients, whilst there were 103 patients in the 2024 cohort. The demographic data of participants are demonstrated in Table 1. The mean age of participants was 51.55 years in the 2019 cohort and 49.49 years in the 2024 cohort (range 18-89). The male-to-female ratio was 45:5 in 2019 and 33:7 in 2024.

**Table 1:** Baseline demographics of patients attending the orthopaedic fracture clinic

Domain		2019 Cohort	2024 Cohort	p-value
Gender	Male	45 (46.0%)	33 (32.03%)	0.1084
	Female	53 (54.0%)	70 (67.96%)	
Age (Years)	<35	20 (20.20%)	47 (45.63%)	-
	35-44	21(21.42%)	15 (14.56.%)	-
	45-54	16 (16.33%)	12 (11.65%)	-
	55-64	15 (15.31%)	20 (19.42%)	-
	65-74	8 (8.16%)	12 (11.65%)	-
	>75	18 (18.37%)	16 (15.53%)	-
	Mean ± Standard Deviation	51.55 ± 19.82	49.49 ± 21.57	-
Employment Status	Higher Education	3 (3%)	12 (11.65%)	-
	Unemployed	7 (7%)	6 (5.82%)	-
	Part-Time Employed	15 (15%)	14 (13.59%)	-
	Full-Time Employed	44 (45%)	40 (38.83%)	-
	Retired	29 (30%)	31 (30.09%)	-
Ethnicity	British	81 (83.51%)	75 (72.81%)	-
	African	5 (5.15%)	2 (1.94%)	-
	Asian	3 (3.09%)	9 (8.73%)	-
	Arabic	1 (1.03%)	5 (4.85%)	-
	White and Black Caribbean	1 (1.03%)	6 (5.82%)	-
	White and Asian	1 (1.03%)	1 (0.09%)	-
	Any Other White Background	1 (1.03%)	3 (2.91%)	-
	Other	4 (4.12%)	2 (1.94%)	-

Previous missed appointments

**Figure 1:**
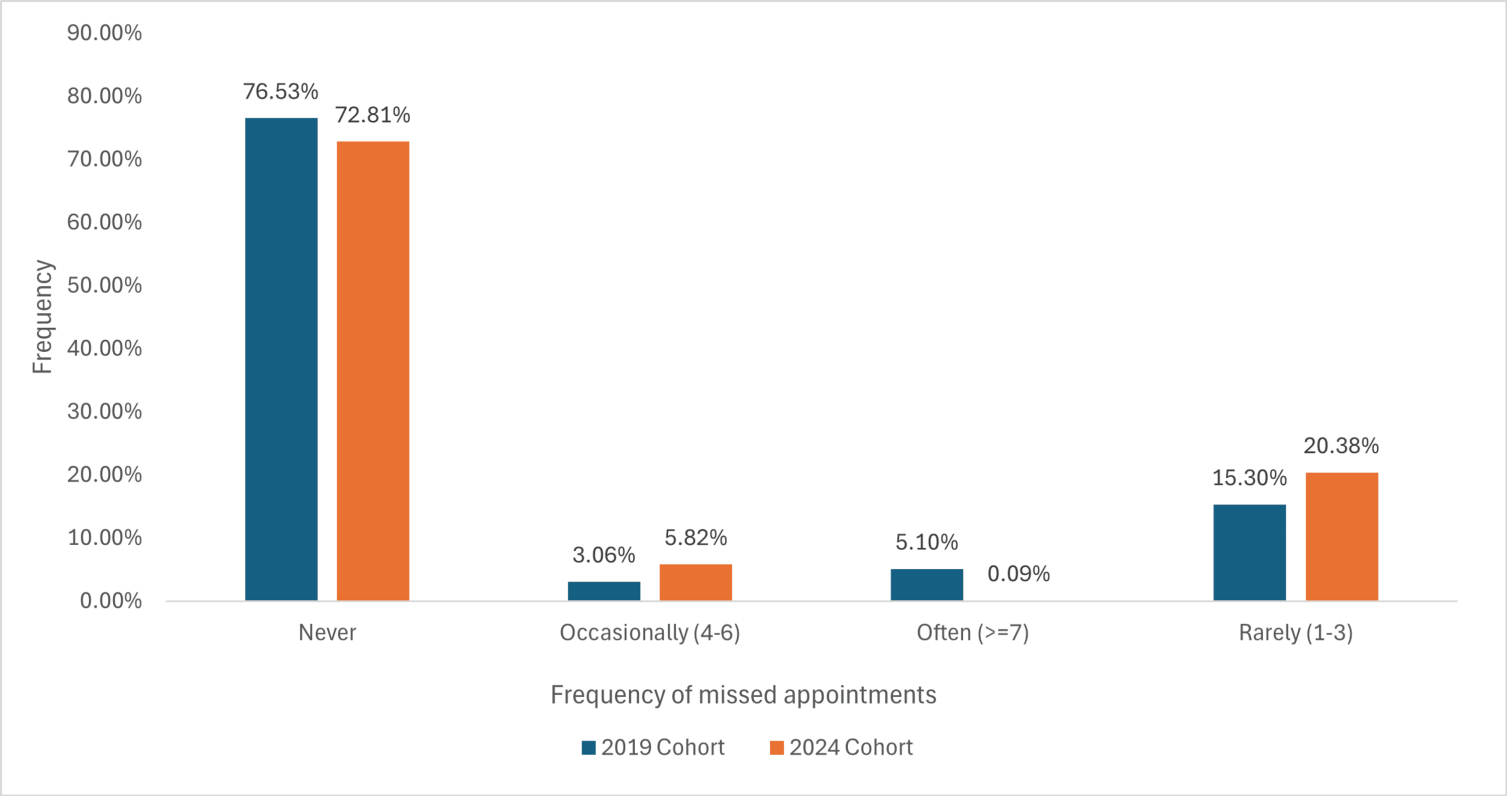
A graph demonstrating the percentage of missed appointments between two cohorts attending an orthopaedic fracture clinic

Ninety-eight responses in the 2019 cohort, whilst 103 in the 2024 cohort, were recorded about previously missed appointments outlined in Figure 1.

The frequency of missed appointments shows similar trends between the 2019 and 2024 cohorts. The majority of participants reported never missing appointments in both cohorts: 75 (76.53%) in 2019 and 75 (72.81%) in 2024. Participants who missed appointments ‘rarely’ (one to three times) showed a slight increase from 15 (15.30%) in 2019 to 21 (20.38%) in 2024. Meanwhile, those who missed appointments ‘occasionally’ (four to six times) remained low but increased slightly from three (3.06%) to six (5.82%). Notably, the proportion of individuals who missed appointments ‘often’ (≥7 times) decreased from five (5.10%) in 2019 to one (0.09%) in 2024. These findings suggest relatively stable patterns of missed appointments across the two cohorts, with a marginal reduction in frequent missed appointments over time.

Access to technology

**Figure 2:**
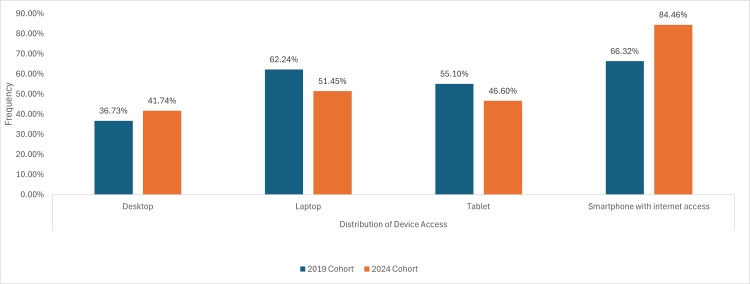
A graph demonstrating device ownership between cohorts attending an orthopaedics fracture clinic

In the 2019 cohort, 65 (66.32%) of participants owned a smartphone with access to the Internet, which increased to 87 (84.46%) in the 2024 cohort. Among participants, 47 (45.63%) had access to a laptop, 47 (43.68%) to a tablet, and 39 (37.86%) had a desktop, as shown in Figure 2.

Ownership of email

**Figure 3:**
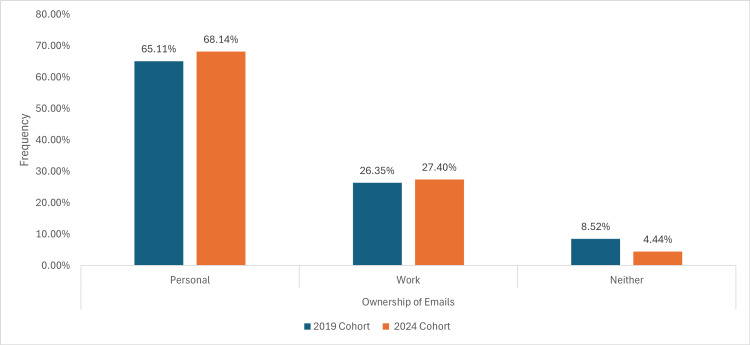
A graph demonstrating the breakdown of the percentage of patients who have active email accounts

The majority of participants in both cohorts had at least a personal or work email. The percentage of people having a personal email rose from 84 (65.11%) to 92 (68.14%) from the 2019 cohort to the 2024 cohort, whilst for work email, from 34 (26.35%) to 37 (27.40%). The proportion of respondents lacking a personal or work email decreased from 11 (8.52%) to six (4.44%), as shown in Figure 3.

Distribution of checking email frequency

**Figure 4:**
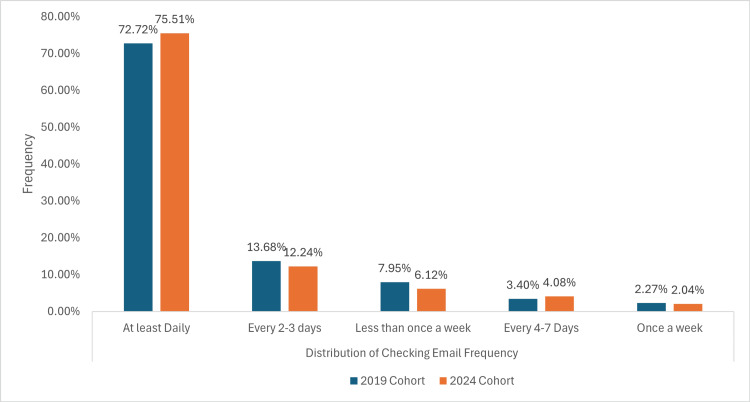
A graph demonstrating how often patients check their emails

In both cohorts, most participants check their email daily, with 64 (72.72%) in the 2019 cohort and 74 (75.51%) in the 2024 cohort, as shown in Figure 4.

Around a similar number of individuals, 12 (13.63%) in 2019 and 12 (12.24%) in 2024 checked their email every two to three days in both cohorts. The number of participants checking emails less than once a week, every four to seven days, and once a week remains similar between the two groups, with minor variations, with no statistically significant difference between the two cohorts (p = 0.9818). In 2019, 78 (84.78%) of participants had email availability on internet-enabled mobile devices, whilst the number increased to 90 (89.10%) in the 2024 cohort.

Satisfaction with letter-based appointment

**Figure 5:**
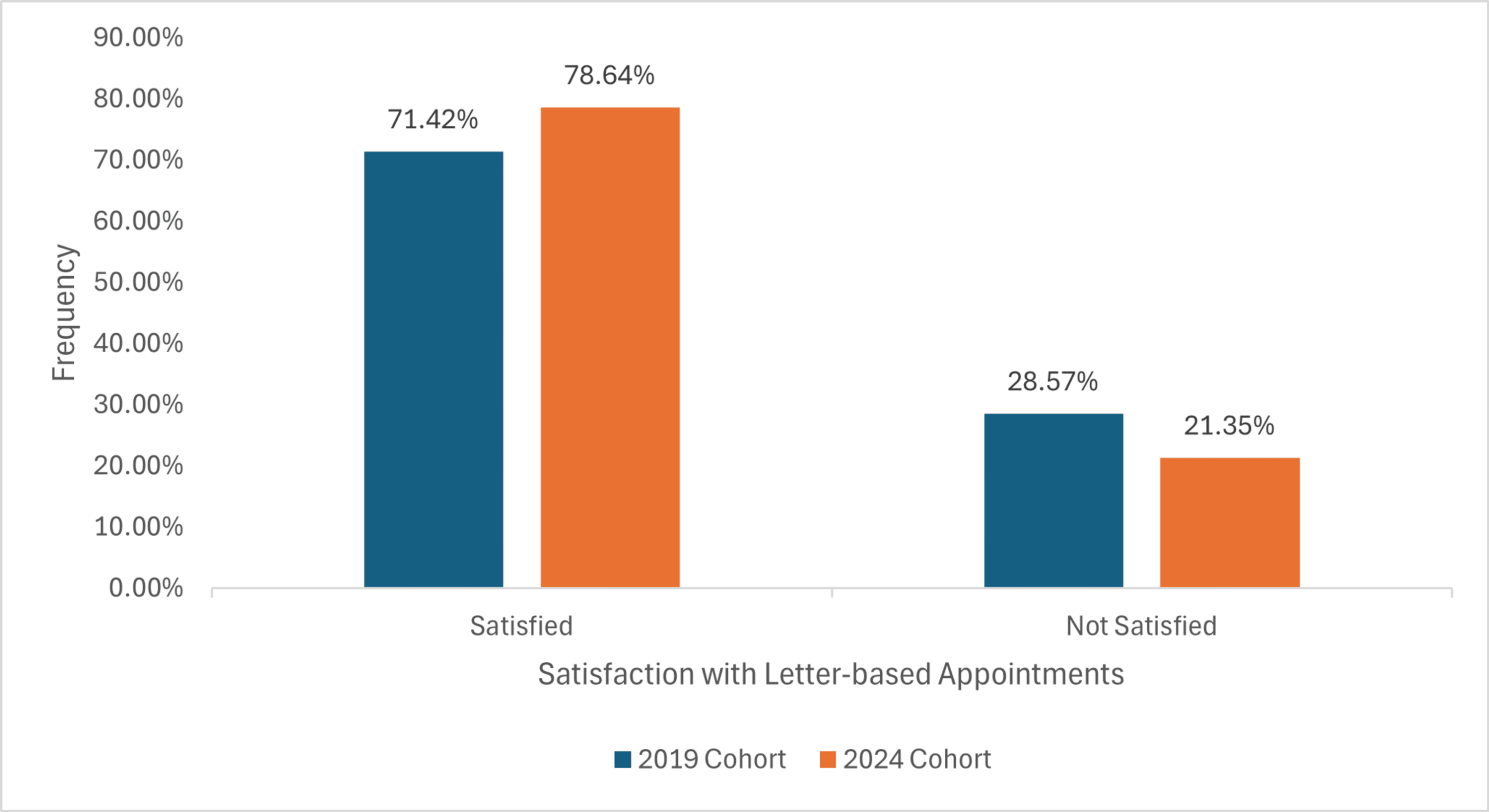
A graph demonstrating satisfaction with letter-based appointments

In the 2019 cohort, 70 (71.42%) reported satisfaction with letter-based appointments, whilst, in the 2024 cohort, 81 (78.64%) expressed satisfaction, with a p-value of 0.308, suggesting no statistical significance, as illustrated in Figure 5.

**Figure 6:**
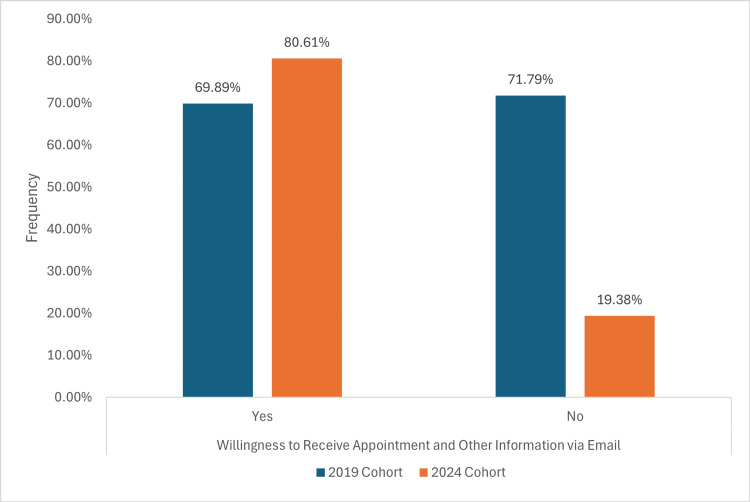
A graph demonstrating a willingness to transition to email-based appointment notifications

On the contrary, in the 2019 cohort, 65 (69.89%) preferred receiving appointments via email, whereas 79 (80.61%) in the 2024 cohort preferred email notifications with no statistical difference, as depicted in Figure 6.

Email communication from the fracture clinic

**Figure 7:**
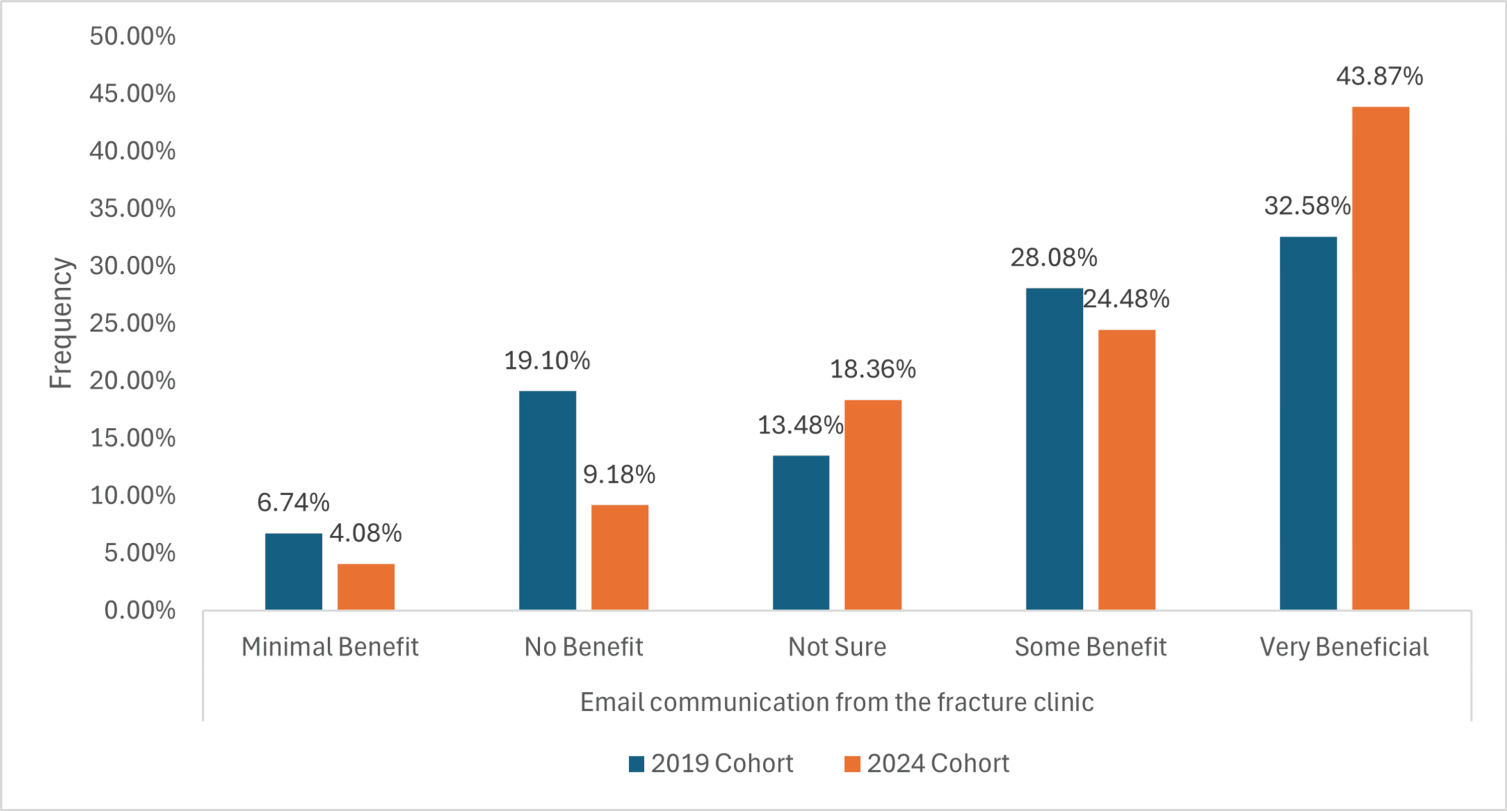
A graph demonstrating how patients perceive and respond to clinic communications sent via email

In the 2019 cohort, six (6.74%) of participants and six (4.08%) of the 2024 cohort reported minimal benefit from email communication, whilst the ‘No Benefit’ category decreased from 17 (19.10%) to nine (9.18%). The ‘Not Sure’ group increased from 12 (13.48%) in the 2019 cohort to 18 (18.36%) in the 2024 cohort. Those reporting ‘Some Benefit’ slightly declined from 25 (28.08%) to 24 (24.48%), but the ‘Very Beneficial’ category rose significantly from 29 (32.58%) to 43 (43.87%), as illustrated in Figure 7.

Privacy concerns regarding email

**Figure 8:**
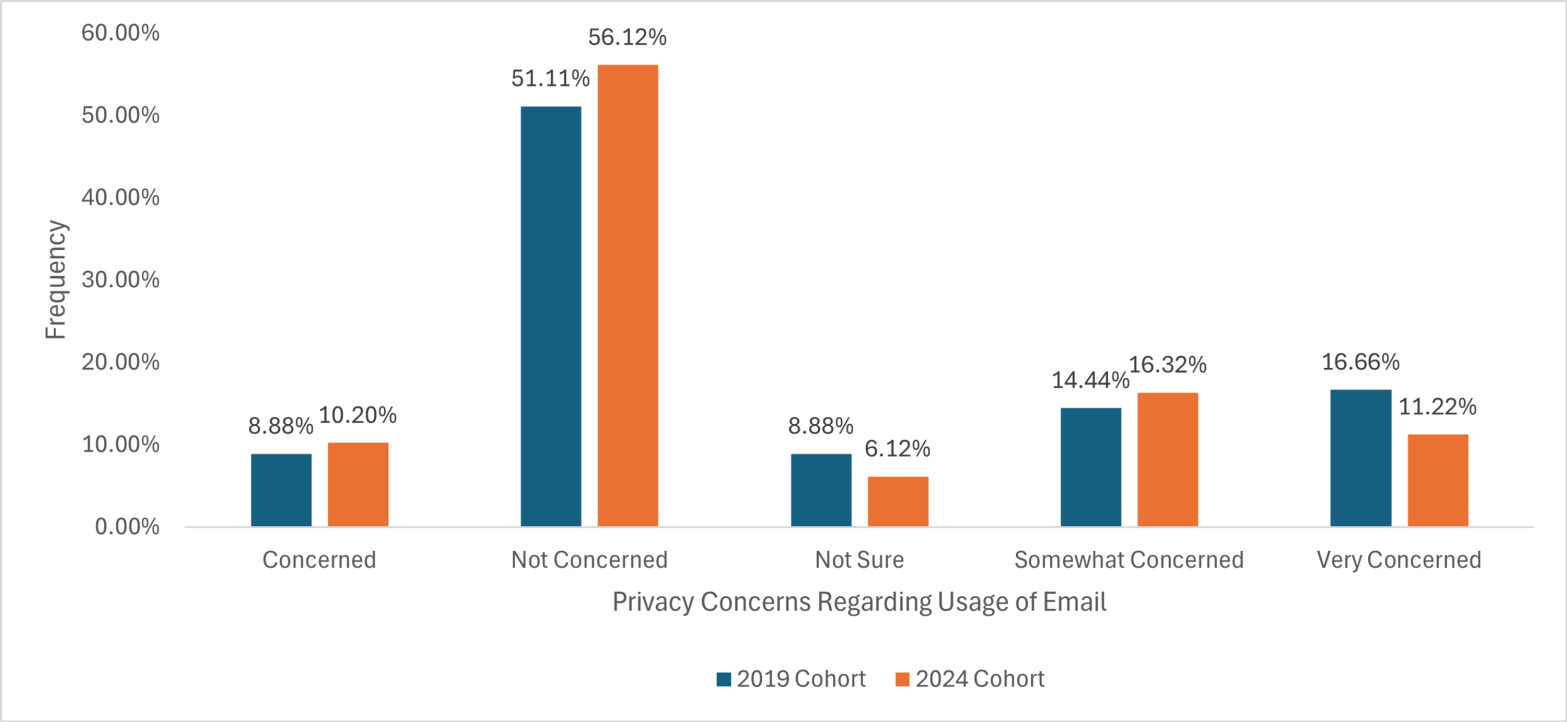
A graph demonstrating patient concerns regarding the security and confidentiality of using email for receiving sensitive medical information

In the 2019 cohort, eight (8.88%) of participants reported being ‘Concerned’ about email privacy, whilst 10.20% expressed the same in the 2024 cohort. The ‘Not Concerned’ category increased slightly from 46 (51.11%) in 2019 to 56.12% in 2024. Those ‘Not Sure’ decreased from 8 (8.88%) to 6 (6.12%), whilst participants who were ‘Somewhat Concerned’ rose from 13 (14.44%) in 2019 to 16 (16.32%) in 2024. The percentage of participants who were ‘Very Concerned’ decreased from 15 (16.66%) to 11 (11.22%), as illustrated in Figure 8.

Junk mail/spam concerns

**Figure 9:**
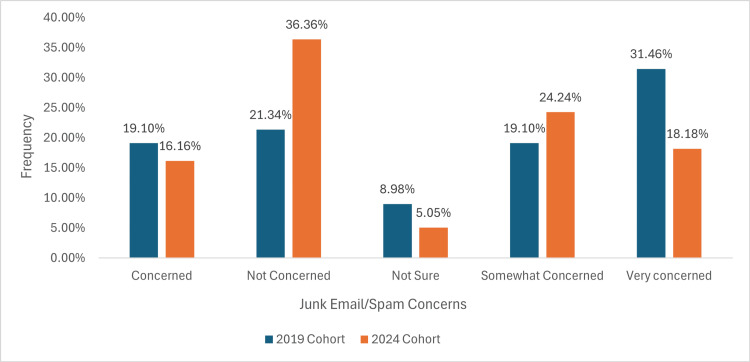
A graph demonstrating patient concerns about important medical emails being misclassified as junk or spam, affecting appointment attendance

In the 2019 cohort, 17 (19.10%) were concerned about junk mail/spam, compared to 16 (16.16%) in 2024. The ‘Not Concerned’ group increased from 19 (21.34%) in 2019 to 36 (36.36%) in 2024, whilst those unsure decreased from eight (8.98%) to five (5.05%). The ‘Somewhat Concerned’ category rose from 17 (19.10%) to 24 (24.24%), and the ‘Very Concerned’ group dropped from 28 (31.46%) to 18 (18.18%) as illustrated in Figure 9.

Ideal communication method

**Figure 10:**
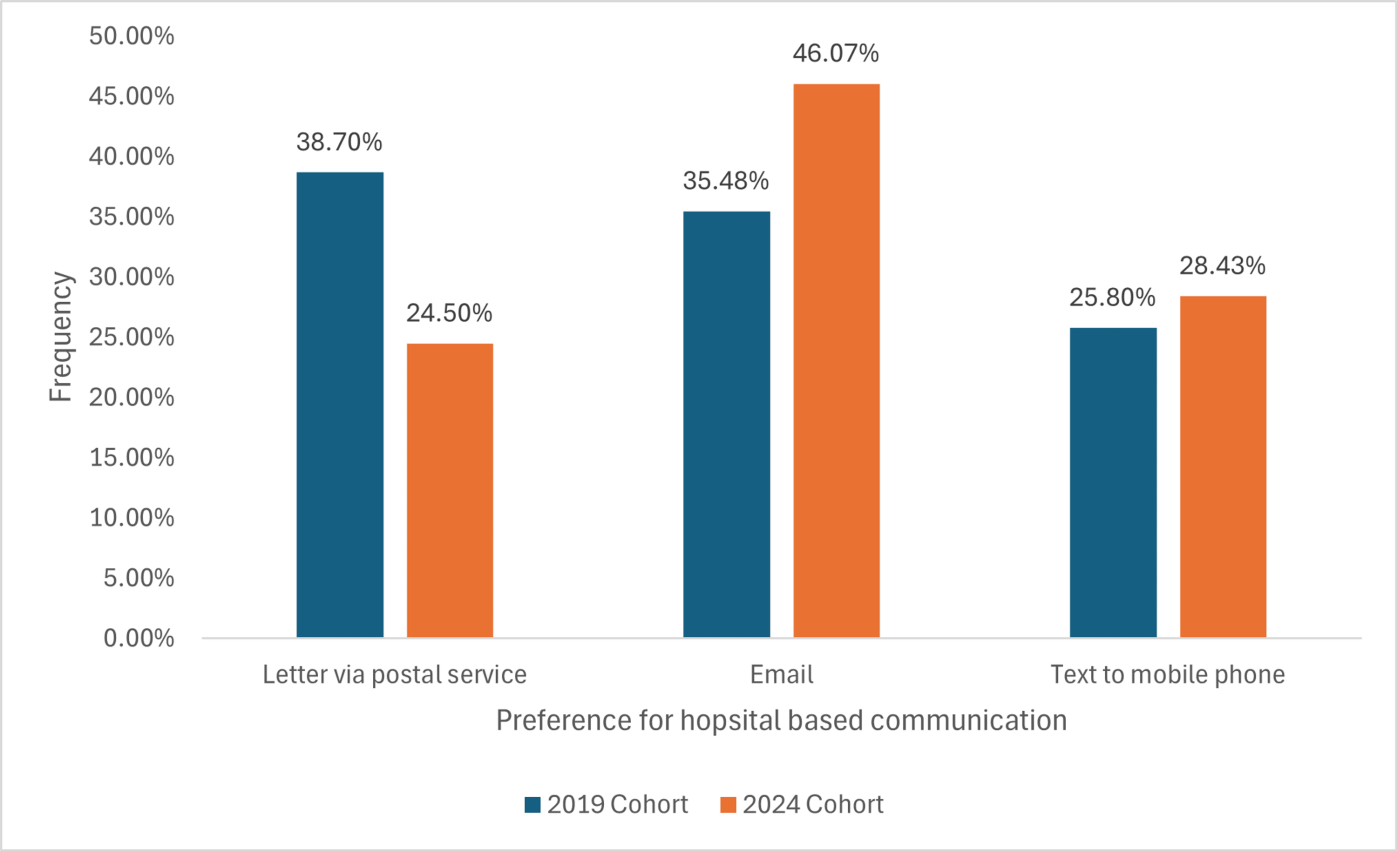
A graph demonstrating the preferred modes of communication, comparing email, phone calls, text messages and postal letters for hospital-based communication between two cohorts

In the 2019 cohort, 36 participants (38.70%) preferred receiving communication via postal letter, whilst this number decreased to 25 (24.50%) in the 2024 cohort. Email preferences rose from 33 (35.48%) in the 2019 cohort to 47 (46.07%) in the 2024 cohort. Similarly, text message preferences showed a slight increase from 24 (25.80%) in the 2019 cohort to 29 (28.43%) in the 2024 cohort (Figure 10).

Preferred email communication for various information categories

**Figure 11:**
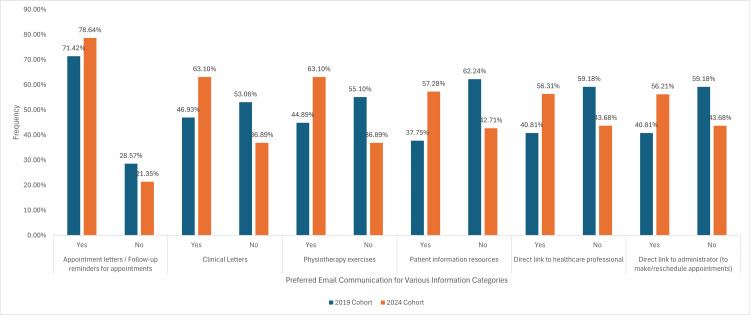
A graph demonstrating preferred healthcare information for email communication: appointment Reminders, test results and follow-up care

For appointment letters and follow-up reminders, 70 (71.42%) of participants in the 2019 cohort preferred email, compared to 81 (78.64%) in the 2024 cohort, with no statistically significant difference. In the case of clinical letters, the percentage increased from 46 (46.93%) in the 2019 cohort to 65 (63.10%) in the 2024 cohort, suggesting a significant difference. Regarding information on physiotherapy exercises, 44 (44.89%) in the 2019 cohort and 65 (63.10%) in the 2024 cohort preferred receiving them over email, with no significant difference. Regarding patient information resources, 37 (37.75%) in the 2019 cohort versus 59 (57.28%) in the 2024 cohort. A significant number of participants (p-value 0.0398) preferred a direct link to healthcare professionals, with a 40 (40.81%) ‘yes’ response in the 2019 cohort and 58 (56.31%) in the 2024 cohort. Finally, for a direct link to administrators for making or rescheduling appointments, the percentage of ‘Yes’ responses was 40 (40.81%) in the 2019 cohort and 58 (56.21%) in the 2024 cohort, with no significant difference, as illustrated in Figure 11.

## Discussion

This study explores patient perspectives regarding their preference for various modes of contact to further aid hospital communications. Comparison of cohorts from 2019 to 2024 displays a shift towards electronic communication, which in the long term could be cheaper due to a reduction in the cost of letter production and delivery, potentially leading to increased attendance of patients in clinics. A shift was seen in a significant number of participants' acceptance of clinic letters, patient information resources and direct links to healthcare professionals from 2019 to 2024. Satisfaction with the current letter-based appointment system was high overall. There was no significant difference in the age group of people missing appointments, and thus, communication preferences should be explored further to understand if DNA rates can be reduced by optimising communication based on patient preference. Multiple studies have reported that the most common reason for DNAs is that patients simply forget [11-13]. Roberts et al. reported there could be a reduction in non-attendance if a text messaging reminder system was implemented [3]. Multiple systematic reviews [5] looking into electronic communication conclude that text messaging and emails do indeed reduce DNA rates [11,12]. In the present study, a significant number of respondents preferred electronic communication of the fracture clinic from the hospital. Electronic communication has not only been shown to be cheaper and faster [14] but may be more suited to the lifestyle needs of a working individual. Through more effective communication means suited to a patient, it may be possible to minimise DNA rates. The majority of new technology further enables an individual to synchronise communications to their digital calendar, which may be further advantageous.

Each communication method has advantages and drawbacks; however, it is evident that digital communication methods have a quicker response time than direct letter communication. This may be attributed to the unconscious pleasure of dopamine release associated with receiving messages via text, which means that 90% of text messages are opened within the first three minutes of delivery [14]. However, digital communication relies on accurate patient data. One study found that patients may enter their phone numbers incorrectly or change their numbers without updating the relevant bodies, leaving any communication sent void [15]. It could be argued that these issues resonate with the current letter communication methods, with addresses changing less regularly or entered incorrectly. Apart from the reasons about familiarity with technology and generational habits, the access to technology in respondents of different age groups may explain this difference in preference. Younger generations were more likely to own portable electronic devices and more frequently check their email, meaning they were likely to read appointment letters or reminders sent via texts or emails. In contrast, over 75 owned fewer portable devices, most likely owning a desktop and checking their emails less frequently [16]. Our survey showed that a majority of respondents, regardless of age, were willing to be contacted about fracture clinic appointments and updates via email. One solution to reduce DNAs, considering the views expressed by this cohort, is to implement a system where the patient can pick which method of communication they prefer for their reminders. A system like this is effective, with a significant reduction of DNA rates seen when patients were contacted using their preferred method of communication [12]. With electronic communication methods costing less than letter-based systems [17], allowing patients to opt for electronic reminders will not only optimise resource allocation within a healthcare system but also reduce the carbon footprint associated with a letter-based system [18,19].

There has also been an evolving trend in privacy concerns with increased email usage over five years. Managing privacy and these concerns will be a significant factor in the potential uptake of email-based reminder systems. Whilst the possibility of confidential information being delivered to unintended recipients is not greater than the current method of communication via letter systems, email encryption and safe data storage can be put in place to minimise any risk associated with email communication [7,20]. Moreover, patients can be educated on what to expect from email communication from a hospital and the dangers of email, such as never giving out their details. It is important to ensure that guidelines for communication with patients are followed to ensure a standard of care is being met and confidentiality upheld using electronic communication methods. This includes monitoring and replying within the appropriate times, responding professionally, avoiding email jargon and communicating only from a secure NHS email address to uphold security and privacy. Addressing these may combat the reservations held by older age groups regarding email communication, therefore, allowing a larger portion of people to shift to digital communication. There is an increased need for further research to explore the impact not only in terms of patient compliance but also financial aspects in shifting towards electronic communication based on patient demographics. The impact of reducing paper letters and the impact on the environment also needs to be researched. Overall, the study highlights the changing trend towards email communication in healthcare and its potential to improve patient attendance.

Limitations

There are important limitations to recognise. The study demographic contained a higher proportion of individuals over 75 years old, limiting the generalizability of the findings based on age. The study was conducted at a single hospital, which limits the applicability of the results to other healthcare settings. The use of convenience sampling, by recruiting participants attending the clinic, may exclude those who miss appointments or have different preferences, further affecting the study's generalizability. Additionally, reliance on self-reported data regarding missed appointments or technology ownership introduces potential bias. The study's limited age range focused primarily on working-age adults and retirees, potentially overlooking the preferences of younger demographics. Regarding communication methods, the study only explored preferences for letters, text messages and email, excluding other possible channels such as phone calls or online portals. Furthermore, the study does not consider the potential challenges of implementing new communication methods, such as patient access to technology or staff training.

## Conclusions

The study found a shift in patient perception towards electronic communication, especially email, compared over five years (2019-2024). Patients had increased preferences for electronic communication in various categories, such as appointment reminders, clinic letters and patient information. There was an overall increase in access to higher technology, specifically email. Hospitals should consider offering patients the option to receive online communication via email in addition to traditional paper letters.

In view of these findings, hospitals must consider expanding their communication options to include email as a primary method of contact alongside traditional paper letters. This shift could enhance patient satisfaction by offering more convenient, accessible and efficient ways to receive important health information. By aligning with patient preferences and technological advancements, healthcare institutions can improve overall communication, ensuring that it meets the evolving needs of their patient population.

## Appendices

**Table 2:** Service evaluation survey on fracture clinic communication in trauma and orthopaedics Credits: Siddhartha Murhekar, Ali Soffar, Abdullah Khawaja, Aman Patel, Haritha Haridas Mandoth Veetil, Keval Patel, Thomas Lorchan Lewis

Question	Options/Details
Age	-
Gender	Male/Female
Employment Status	Full-Time Employed/Part-Time Employed/Not Currently Employed/Student/Higher Education/Retired
Ethnic Group or Background	-
White	English/Welsh/Scottish/Northern Irish/British/Irish/Gypsy or Irish Traveller/Any Other (Describe)
Mixed/Multiple Ethnic Groups	White and Black Caribbean/White and Black African/White and Asian/Any Other (Describe)
Asian/Asian British	Indian/Pakistani/Bangladeshi/Chinese/Any Other (Describe)
Black/African/Caribbean/Black British	African/Caribbean/Any Other (Describe)
Other Ethnic Group	Arab/Any Other (Describe)
Access to Information and Communication Technologies (ICT) Devices	Desktop PC/Laptop/Tablet/Smartphone With Internet Access
Email Access	Personal Email/Work Email/Neither
Can You Recall Your Email Address Without Looking It Up?	Yes/No
Frequency of Checking Email	At Least Daily/Every Two to Three Days/Every Four to Seven Days/Once a Week/Less Than Once a Week
Is Your Email Available on Your Mobile Device?	Yes/No
Willingness to Receive Appointment Information via Email	Yes/No
Perceived Benefit of Email Communication	No Benefit/Minimal Benefit/Not Sure/Some Benefit/Very Beneficial
Frequency of Missed Hospital Appointments (Due to Postal delays)	Never (0)/Rarely (1-3)/Occasionally (4-6)/Often (≥7)
Concern About Email Privacy	Not Concerned/Somewhat Concerned/Not Sure/Concerned/Very Concerned
Concern About Receiving Junk Mail or Spam	Not Concerned/Somewhat Concerned/Not Sure/Concerned/Very Concerned
Satisfaction With Current Contact Methods	Very Dissatisfied/Somewhat Dissatisfied/Not Sure/Satisfied/Very Satisfied
Preference for Receiving Hospital Communication	Letter via Postal Service/Email/Text to Mobile Phone/Other (Specify)
Useful Information to be Communicated via Email	Appointment Letters/Clinical Letters/Physiotherapy Exercises/Patient Information/Links to Healthcare Professionals or Administrators/Other (Specify)
